# Neurotrophin Gene Therapy for Sustained Neural Preservation after Deafness

**DOI:** 10.1371/journal.pone.0052338

**Published:** 2012-12-17

**Authors:** Patrick J. Atkinson, Andrew K. Wise, Brianna O. Flynn, Bryony A. Nayagam, Clifford R. Hume, Stephen J. O’Leary, Robert K. Shepherd, Rachael T. Richardson

**Affiliations:** 1 Bionics Institute, East Melbourne, Victoria, Australia; 2 Department of Otolaryngology, University of Melbourne, East Melbourne, Victoria, Australia; 3 Department of Medical Bionics, University of Melbourne, East Melbourne, Victoria, Australia; 4 Department of Otolaryngology-Head and Neck Surgery, University of Washington, Seattle, Washington, United States of America; Medical University Innsbruck, Austria

## Abstract

The cochlear implant provides auditory cues to profoundly deaf patients by electrically stimulating the residual spiral ganglion neurons. These neurons, however, undergo progressive degeneration after hearing loss, marked initially by peripheral fibre retraction and ultimately culminating in cell death. This research aims to use gene therapy techniques to both hold and reverse this degeneration by providing a sustained and localised source of neurotrophins to the deafened cochlea. Adenoviral vectors containing green fluorescent protein, with or without neurotrophin-3 and brain derived neurotrophic factor, were injected into the lower basal turn of scala media of guinea pigs ototoxically deafened one week prior to intervention. This single injection resulted in localised and sustained gene expression, principally in the supporting cells within the organ of Corti. Guinea pigs treated with adenoviral neurotrophin-gene therapy had greater neuronal survival compared to contralateral non-treated cochleae when examined at 7 and 11 weeks post injection. Moreover; there was evidence of directed peripheral fibre regrowth towards cells expressing neurotrophin genes after both treatment periods. These data suggest that neurotrophin-gene therapy can provide sustained protection of spiral ganglion neurons and peripheral fibres after hearing loss.

## Introduction

Hearing loss is the most common sensory deficit in developed countries, with an estimated 278 million people globally suffering from a disabling hearing impairment [1], [2]. This number is predicted to rise as the population ages. The most common cause of hearing impairment, sensorineural hearing loss (SNHL), results from severe damage to or loss of cells within the organ of Corti (OC), in particular the sensory hair cells (HCs) and/or the primary neurons, commonly called spiral ganglion neurons (SGNs). Hair cell loss, can result from a number of factors including aging, overexposure to noise, genetic disorders and administration of ototoxic drugs (for example, aminoglycoside antibiotics). In the most severe cases of SNHL the only clinical treatment currently available is a cochlear implant (CI), which electrically stimulates the SGNs via an electrode array located in the scala tympani [3]. However, the loss of HCs and supporting cells results in an ongoing degeneration of SGNs [4], [5], [6], [7], [8], [9], [10], reducing the number of SGNs available for stimulation by a CI. Degeneration of the SGNs is thought to be primarily due to a loss of trophic support normally provided by the HCs and the supporting cells. Neurotrophins (NTs), in particular neutrophin-3 (NT3) and brain derived neurotrophic factor (BDNF), have been shown to play key roles in both the development and survival of SGNs [11], [12], [13], [14], [15], [16], and as such have been the focus of research aiming to mitigate degeneration of SGNs after deafness.

The administration of exogenous NT3 and/or BDNF to the deafened guinea pig (GP) cochlea via a mini-osmotic pump has been shown to promote SGN survival and peripheral fibre regrowth [17], [18], [19]. However, the duration of exogenous NT delivery by a mini osmotic pump is finite, and the protective effect, with NTs alone, has not been shown beyond 2 weeks after cessation of NT administration [19], [20], which suggests that a long-term source is needed for prolonged neural survival. As such, the pump would need to be continually refilled with NTs increasing the risk of infection, which precludes their use as a clinical treatment. Moreover, while NTs delivered via a mini osmotic pump promoted peripheral fibre resprouting, fibre regrowth was disorganised [18], [21], [22], possibly due to the high concentration of NTs infused and their diffusion throughout the cochlea [23]. The disorganised resprouting is characterised by the fibres looping back within the osseous spiral lamina and projecting laterally along the basilar membrane. This is in stark contrast to the highly organised radial projections found in the normal cochlea [18], and as such may degrade the spectral information provided by the implant as a consequence of the spread of neural activation.

**Table 1:** Summary of experimental groups.

Hearing Status	Gene Transfer Vector	Post-injection Treatment Period	N =	Naming Convention
**Normal**	Ad-GFP	11 weeks	5	(T11-wk)
**1 Week Deafened**	N/A	N/A	5	(T1-wk)
**1 Week Deafened**	Ad-GFP	7 weeks	5	(T8-wk)
**1 Week Deafened**	Ad-GFP-NT	7 weeks	5	(T8-wk)
**1 Week Deafened**	Ad-GFP	11 weeks	5	(T12-wk)
**1 Week Deafened**	Ad-GFP-NT	11 weeks	4	(T12-wk)

Gene therapy may be able to address the aforementioned issues associated with direct infusion of NTs into the cochlea, by providing both a long-term and localised source of NTs. Previous studies have indeed shown that the introduction of NTs into the deafened GP cochlea through the use of Adenoviral (Ad) viral vectors encoding for BDNF and/or NT3 promoted SGN survival for up to 4 weeks post treatment [24], [25]. Furthermore, the localised introduction of Ad encoding for NTs into the scala media (SM) has been shown to result in the transduction of the OC, providing directional cues for resprouting peripheral fibres in addition to promoting SGN survival [26], [27]. Although these results are promising, for this treatment to be clinically relevant there needs to be cells within the OC after deafness which are able to be transduced and remain transduced. A recent study showed that the efficacy of NT-gene therapy diminished as the time between deafness onset and intervention increased, highlighting the importance of early intervention [28]. The stability of transduced cells and the corresponding resprouting fibres is also unknown.

In order to be effective, sustained and safe, the survival-promoting effects of a single viral mediated NT delivery would need to be demonstrated over long treatment periods. This study, therefore, aimed to examine the ability of Ad-mediated NT transfection 1 week after deafness onset to provide a sustained, localised and safe source of NTs for long-term SGN rescue and directed regrowth of resprouting peripheral fibres.

**Figure 1:**
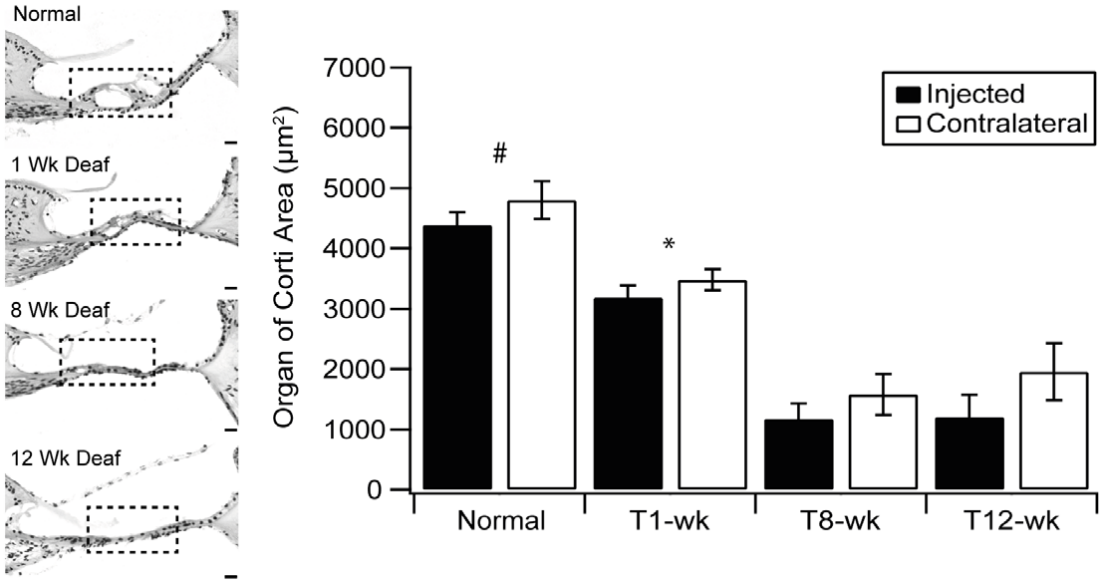
Degeneration of the OC post aminoglycoside deafening. The OC (outlined by black dashed box) degenerated rapidly in the basal turn after aminoglycoside deafening in the GP as illustrated in the representative photomicrographs of the upper basal turn at various time points post deafening. There was a significant difference between the OC area of normal hearing GPs and all other cohorts (#p<0.001, ANOVA). This indicates that even after 1 week significant degeneration had occurred. There is also a significant difference between 1 week and 8 and 12 weeks post deafening (*p<0.001, ANOVA), however, there was no further change after between 8 and 12 weeks. There was no difference in the time course of degeneration between left (NT-treated) cochleae and right (non-treated cochleae). These data gives an indication of the status of the OC at the time of gene therapy intervention (1 Wk Deaf) and at the times of analysis (8 Wk and 12 Wk Deaf). Error bars indicate the standard error of the mean (n = 4–5 GPs per point). Scale bars = 20 µm.

## Materials and Methods

### Ethics Statement

National Health and Medical Research Council and National Institutes of Health (NIH) Guidelines for the Care and Use of Laboratory Animals were observed. The Animal Research Ethics Committee of the Royal Victorian Eye and Ear Hospital approved the care and use of the animals in this study (ethics #09/180AB).

**Figure 2:**
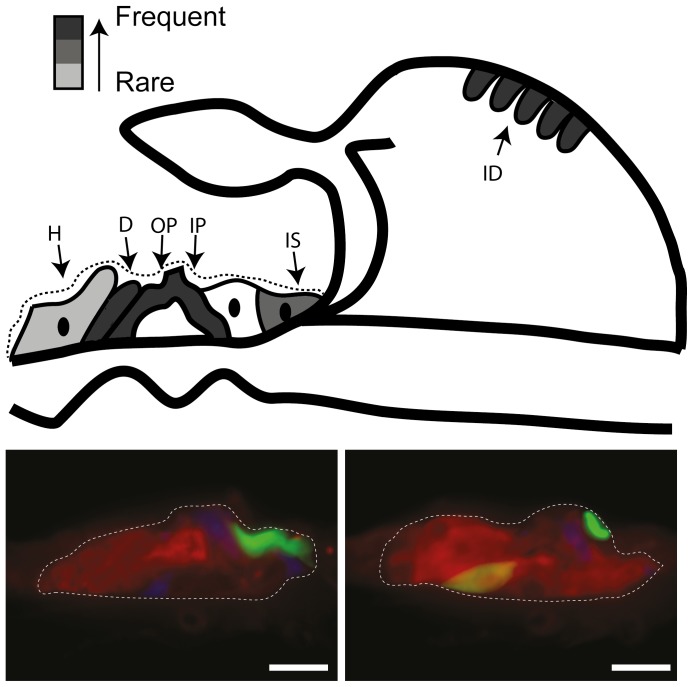
Schematic and photomicrographs of GFP expression in the deafened GP cochlea. Injection of Ad-GFP or Ad-GFP-NTs into the SM when examined after at T12-wk (n = 9) resulted in GFP expression in the OC and interdental cells of the spiral limbus of the lower basal and upper basal turns (represented by greyscale coded schematic, with dark grey representing areas where GFP expression was frequently observed (n>5) and light grey representing areas where GFP expression was only rarely observed (n<2). Photomicrographs illustrate GFP expression (green), in the OC, specifically the inner and outer pillar cells (IP, OP), along with Deiters’ (D), Hensen’s (H) cells, inner sulcus cells (IS) and the interdental cells (ID) of the spiral limbus. Sections are co-labelled with anti-calretinin (red) and phalloidin (blue). Scale bars = 20 µm.

### Ad Vectors

E1/E3/polymerase/terminal protein-deleted Ad type 5 vectors containing GFP under the control of a cytomegalovirus promoter with or without mouse NT3 or BDNF expressed via an internal ribosome entry site sequence (Ad-GFP, Ad-GFP-NT3, and Ad-GFP-BDNF) were generated using the AdEasy system (Stratagene, La Jolla, CA) as detailed previously [26]. In vitro testing of Ad-GFP-NT3 and Ad-GFP-BDNF confirmed release of neurotrophins from infected cells by ELISA [26]. Prior to injection, Ad vectors were diluted 1∶5 in artificial endolymph (120 mmol/l KCL, 2.5 mmol/l NaCl, 0.5 mmol/l MgCl_2_, 028 mmol/l CaCl_2_, 7.6 mmol/l K_2_HPO_4_, 2.7 mmol/l KH_2_PO_4_, pH 7.4) to final concentrations of 1.1×10^11^ OPU/ml (Ad-GFP), 3.0×10^10^ OPU/ml (Ad-GFP-NT3) and 4.33×10^10^ OPU/ml (Ad-GFP-BDNF). Ad-GFP-NT3 and Ad-GFP-BDNF were mixed in a 1∶1 ratio just prior to injection and will hereon be referred to as Ad-GFP-NTs.

### Animals and Ethics

Male or female adult pigmented Dunkin-Hartley GPs (n = 29, average weight 398±16.36 g) were used in this study. Viral administration, when necessary, was performed with the approval of the Office of the Gene Technology Regulator Australia (licence #444). GPs were randomly assigned to experimental groups described in Table 1.

### ABR Recordings

#### Deafened groups

The hearing status of each GP in deafened groups was assessed prior to deafening using computer-generated click stimuli and auditory brainstem response (ABR) recordings [29], [30]. For inclusion in the study, GPs were required to have normal hearing prior to deafening (defined as having an ABR threshold <43 dB peak-equivalent sound pressure level).

**Figure 3:**
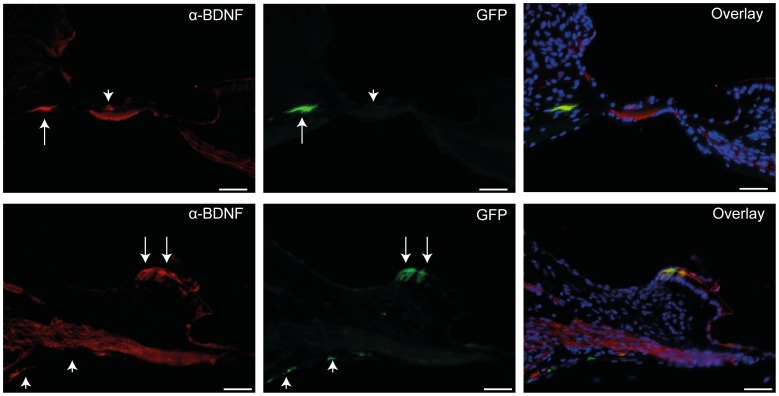
BDNF expression in deafened GPs injected with Ad-GFP-NT at 11 weeks. Two examples (top and bottom) showing anti-BDNF antibody staining (red) co-localised with GFP expression (green) in some (arrows), but not all cases (arrowheads). As GPs were injected with a combination of vectors encoding for BDNF and NT3, not every GFP-positive cell would be expected to co-localise with BDNF staining, as illustrated in overlays above. Scale bars = 50 µm.

#### Normal-hearing group

ABR thresholds from normal-hearing GPs were assessed to determine the effects of the injection technique on hearing thresholds. Tone-pip ABR thresholds (at frequencies of 1, 2, 8, 16, 24 and 32 kHz) [31] were performed at 11 weeks post injection.

### GP Deafening

Normal-hearing GPs were deafened at time 0 (T0-wk) under gaseous anaesthesia via intravenous infusion of 100 mg/kg frusemide (Troy Laboratories, Smithfield, Australia) and subcutaneous 400 mg/kg kanamycin sulphate (Applichem, Taren Point, Australia) [29]. This deafening technique produces a reliable threshold shift of >50 dB [29] and a loss of 80–100% HCs within 7 days, with any remaining HCs restricted to apical cochlear regions [32], [33].

### Cochlear Injection of Viral Sample

Viral vectors (either Ad-GFP-NTs or Ad-GFP) were unilaterally injected into cochleae at T1-wk as previously described [26]. Briefly, the head was secured using an atraumatic head holder under anaesthesia (60 mg/kg ketamine and 4 mg/kg xylazine, intramuscular). A retroauricular incision was made to expose the bulla. After drilling through the bulla, the cochlea was located and a small cochleostomy was made into the otic capsule of the basal turn, using a 2-mm diamond tip drill bit. Perilymph was removed using gentle suction until the basilar membrane could be visualized. A glass recording micropipette (World Precision Instruments, Sarasota, FL) with a 20–30 µm tip diameter was advanced via a stepper motor through the basilar membrane. An endocochlear potential was recorded (63.4±4.4 mV n = 28), indicating the SM had been accessed [34]. Two microliters of the viral sample was injected into the SM over 5 minutes and the micropipette was retracted 1 minute later. The cochleostomy was sealed with connective tissue, the bulla sealed with dental cement, and the wound closed with sutures.

**Figure 4:**
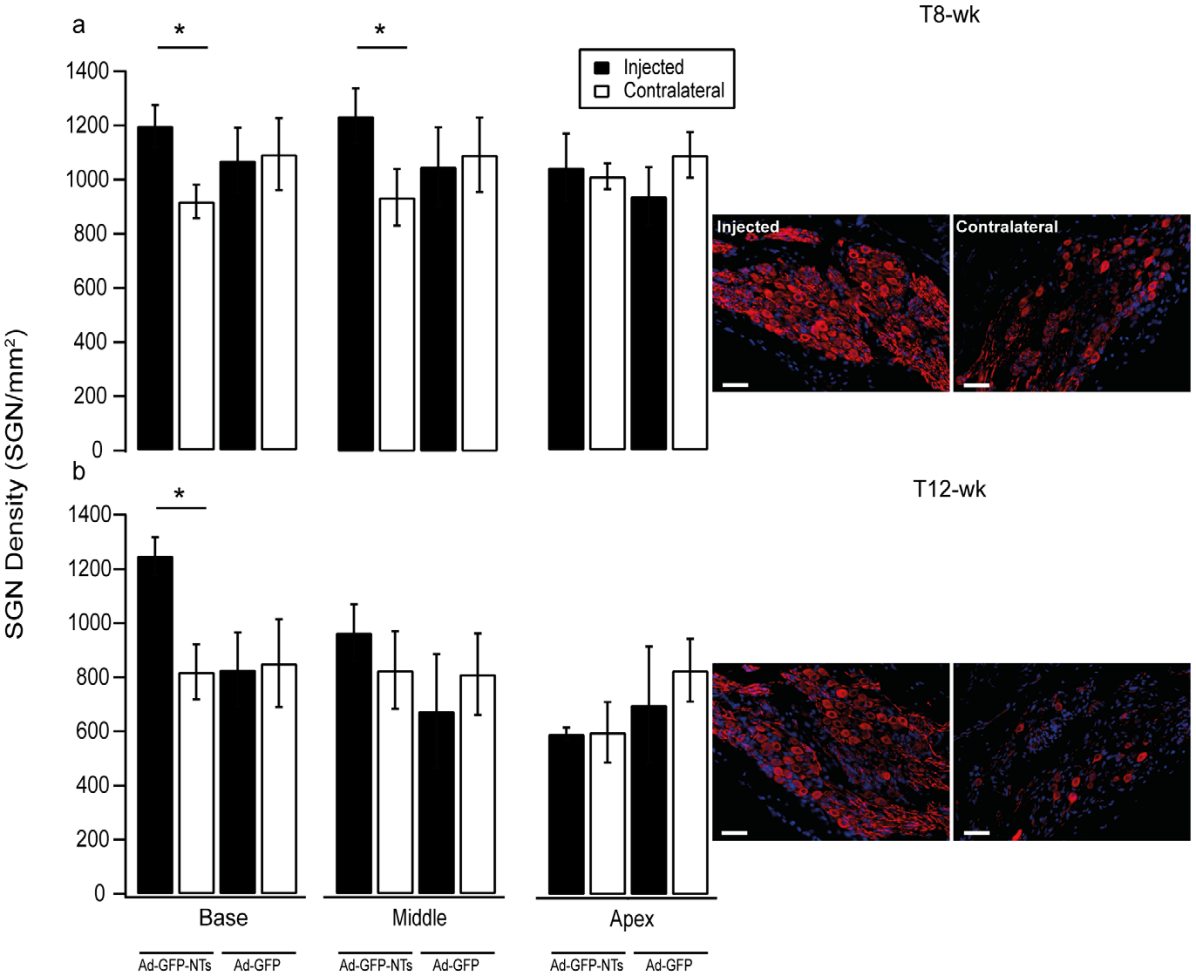
SGN density measurements in treated and untreated cochleae of deafened GPs. SGN density data for deafened cochleae injected with either NTs or GFP control vector and the untreated control cochlea (contralateral) following 7 (a) or 11 (b) weeks treatment. Example photomicrographs of SGNs in the lower basal turn for 7 and 11 week groups that received Ad-GFP-NTs in the injected cochlea. After 7 weeks of treatment there was a significantly greater SGN density in the basal and middle turns of the cochlea (*p<0.05, ANOVA). When examined at 11 weeks post injection there was also significantly greater SGN density in the basal turn of the cochlea (*p<0.05, ANOVA). Error bars indicate the standard error of the mean (n = 4–5 GPs per point). Scale bar = 50 µm.

### Perfusion, Cochlear Sectioning, and Immunohistochemistry

After the appropriate deafness or treatment period (see Table 1) GPs were euthanized with 1.5 ml pentobarbitone and intracardially perfused with 0.9% (wt/vol) saline containing 0.1% (vol/vol) heparin sodium and 0.025% (wt/vol) sodium nitrite, followed by 10% (vol/vol) neutral buffered formalin. The bullae were removed and the cochleae dissected. Cochleae were placed in 10% (vol/vol) neutral buffered formalin for a further 12–16 hours and then decalcified over 3–4 weeks at 4°C days in 10% (wt/vol) EDTA in 0.1 mol/m phosphate buffer. Cochleae were embedded in OCT (Tissue-Tek, Torrance, CA) and sectioned on a cryostat at 12 µm through pre-modiolar and mid-modiolar planes and mounted onto SuperFrost Plus slides (Menzel-Gläser, Braunschweig, Germany), leaving the final half of the cochleae intact. The remaining half-cochleae that contained the viral injection site were cut into half-turn surface preparations [18], [26]. The Reissner’s and tectorial membranes were removed, allowing for better visualisation of the basilar membrane. Standard immunofluorescent protocols were followed using antibodies for neurofilament-200 (1∶200, NF-200; Merck Millipore, Australia) to stain the SGNs and peripheral fibres, anti-calretinin (1∶500, Merck Millipore, Australia) and phalloidin (1∶80, Molecular Probes, USA) to stain the cells in the OC, and antibodies for NT3 and BDNF (1∶100, Santa Cruz Biotechnology, Santa Cruz, CA) and AlexaFluor secondary antibodies (1∶500, Molecular Probes, USA) were used to visualize several antibodies in the same sample. Sections were examined on a Zeiss Axioplan fluorescence microscope (Carl Zeiss, Germany). Cochlear half-turn surface preparations and pre mid-modiolar sections were viewed on a Zeiss Meta confocal microscope.

### Data Analysis

#### GFP expression

The GFP reporter gene was used to assess gene expression in mid-modiolar sections and cochlear half turns. The location of GFP expression from three non-consecutive mid-modiolar sections (over 72 µm) was marked on a representative mid-modiolar image of a GP cochlea to form a composite image.

**Figure 5:**
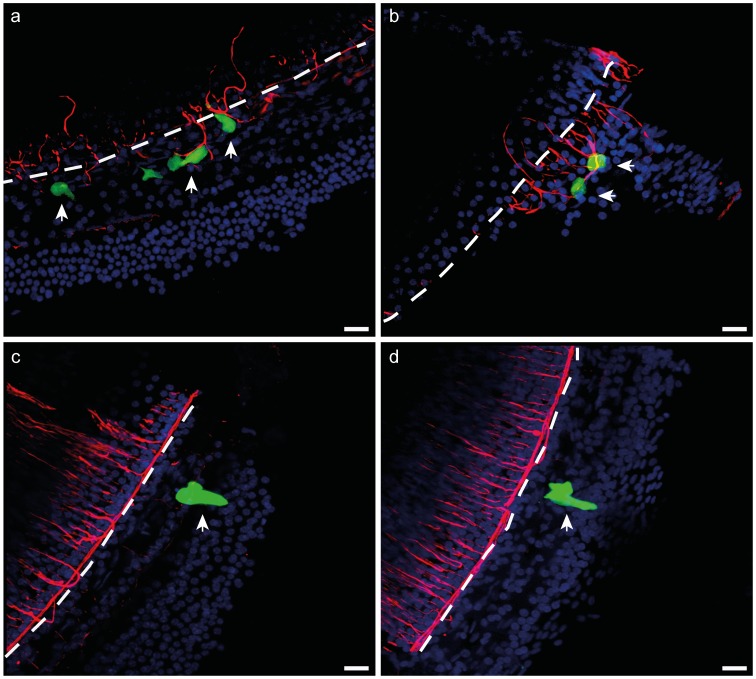
Peripheral fibres growing proximal to Ad-GFP-NTs transduced cells. Cochlear surface preps were stained with anti-NF200 (red) and DAPI (blue). (**a–b**) Peripheral fibres response to neurotrophin-expressing cells (arrows) in the OC 11 weeks post Ad-GFP-NTs treatment. (**c–d**) Peripheral fibre response to cells expressing GFP only (arrows) in the OC 11 weeks post Ad-GFP injection. Significantly greater densities of fibres were observed around NT-secreting cells compared to GFP-only transduced cells (p<0.05, t-test). The dotted line indicates the inner edge, the approximate site of the inner pillar cells of the OC. Scale bar = 50 µm.

#### SGN survival and OC degeneration

SGN density was analysed from three non-consecutive (over 72 µm) mid-modiolar sections from each cochlea in a blinded manner. Density was determined by counting NF-200 and DAPI-positive SGN cell bodies within Rosenthal’s canal and by measuring the area of Rosenthal’s canal using ImageJ software (NIH, USA). Lower basal and upper basal SGN densities were averaged to calculate SGN density in the basal turn. The same technique was used for middle and apical turns. Statistical analyses of SGN density data were performed using repeated-measures one way ANOVA and a post hoc Holm-Sidak test.

To determine whether NT gene therapy had an effect on the size of the surviving SGNs the soma area of SGNs was measured in the Ad-GFP-NT treated and non-injected contralateral cochleae from the T8-wk- and T12-wk treatment groups. Soma area was measured, in a blinded manner, in the lower and upper basal region by randomly selecting 10 NF-200 and DAPI-positive SGNs in three non-consecutive mid-modiolar sections. A grid and a random number generator were used for the randomised selection process. The outer perimeter of the soma was traced in Image J (NIH, USA) and the area calculated. Mean area (± SEM) was determined for a total of 30 SGNs for the treated and un-treated contralateral cochlea, and evaluated using a paired t-test.

The progressive degeneration of the OC following aminoglycoside exposure was reported to reduce gene transduction when applied after prolonged deafness [28]. Therefore, to establish the effect of gene therapy on OC degeneration over time, OC area was measured in the injected cochlea and compared to that in the contralateral (control) cochlea. Measurements were based on sections stained with anti-calretinin and phalloidin, allowing for visualisation of the supporting cells within the OC. The upper basal turn of treated and non-treated contralateral control cochleae of each group were measured. A cohort of normal hearing animals was also used for comparison. Area measurements were taken from three non-consecutive mid-modiolar sections (over 72 µm) and presented as mean ± SEM; statistical analysis was carried out using a two-way repeated measures (RM) ANOVA.

#### Quantification of peripheral fibre regrowth

To examine whether localised NT expression within residual cells of the OC would influence peripheral fibre growth over long durations following a hearing loss, the density of peripheral fibres in close proximity to transduced cells was examined in confocal images of cochlear surface preparations. Pixel density occupied by NF-200 labelling was measured within a boundary of 10 µm from the perimeter of the GFP-expressing cells. Only GFP-expressing cells within the OC region and distal to the inner HCs were used for analysis to avoid counting fibres that are normally observed in the inner spiral bundle, as such not all surface preparations could be included in this analysis (T8-wk cohorts: n = 3 GPs for Ad-GFP, n = 2 for Ad-GFP-NTs, and T12-wk cohorts: n = 2 for Ad-GFP, n = 4 for Ad-GFP-NTs). Consecutive Z-planes in which both the GFP-expressing cell and the NF-200 labelling were in the focal plane were averaged. Measurements from Ad-GFP injected GPs and Ad-GFP-NTs injected GPs were compared using a t-test.

**Figure 6:**
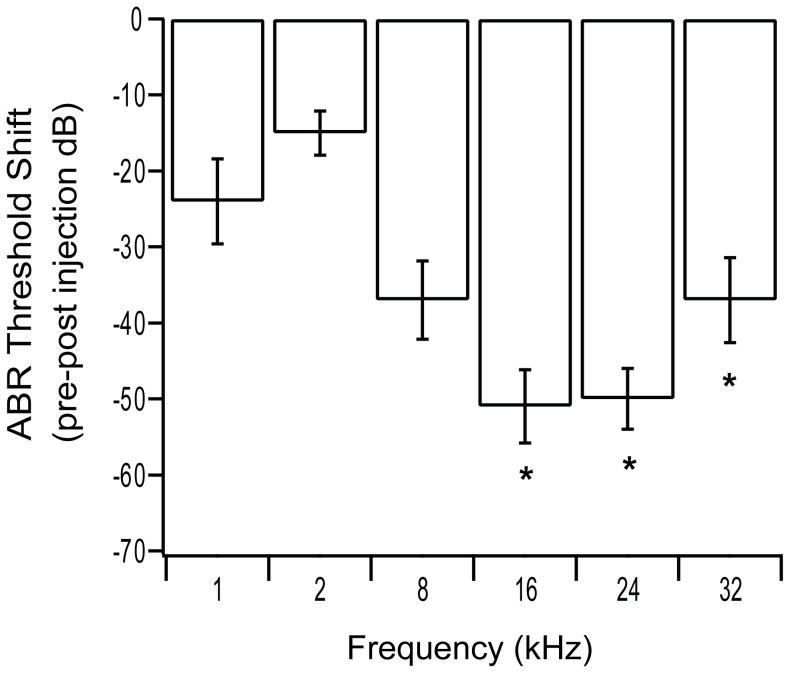
ABR threshold shifts in normal hearing GPs following viral injection. Shifts in ABR thresholds were observed in normal-hearing GPs following injection with Ad-GFP. ABRs were measured at 1 week pre-injection and at 11 weeks post-injection. Significant threshold shifts were observed at the higher frequencies (16–32 kHz) (*p<0.05 paired t-test), the region at which the injection was made. Error bars represent standard error of the mean (n = 5 GPs per bar).

#### Histological analyses

The chronic tissue response to the viral injection surgery was assessed by measuring the area of the tissue response (fibrous tissue and new bone) and calculating that as a percentage of the total area of the scala tympani. Tissue response was quantified in haematoxylin and eosin-stained sections in three non-consecutive cochlear sections (72 µm apart) within the basal (and when necessary more apical) turns using a Zeiss Axio Imager M2 microscope and analysed using Image J.

## Results

### 1. Gene Expression

#### (a) OC degeneration

As expected, the OC exhibited gradual degeneration over the time course examined as evident in the histological sections in figure 1. Measurement of the area of the upper basal OC reveal there was a significant main effect of duration of deafness on the area of the OC (two way repeated measures ANOVA, p<0.001). Post hoc analysis showed a statistical difference between the normal hearing animals and all other time points post deafening, moreover there was a significant difference between one and eight weeks post deafening (two way repeated measures ANOVA, post hoc Holm-Sidak, p<0.001). There was no difference between eight and twelve weeks post deafening. Interestingly, there was no difference in the degeneration time course between NT-treated cochleae and contralateral non-treated cochleae.

#### (b) Viral vector expression profile

As reported in previous studies [26], [28], there was no discernible difference in the types of cells transduced or the spatial extent of expression between Ad-GFP and Ad-GFP-NT groups in mid-modiolar sections and basal turn surface preparations. The GFP expression data were therefore combined and a schematic representation of GFP distribution is shown in figure 2. Despite the partial degeneration of the OC at the time of injection (T1-wk), transduction of cells within the OC was possible and in all cases GFP expression was detected in the basal turn of the cochlea, the area most proximal to the site of injection. GFP was observed in the middle turn in 7 out of 10 treated cochleae examined at T8-wk and in 4 out of 9 of those examined at T12-wk. Expression was only rarely (2 out of 19 cochleae) observed in the apical regions of the cochlea. Expression was most commonly localised to cells within the partially degenerated OC (figure 2), the osseous spiral lamina and the interdental cells. The transduced cells of the OC were identified as the inner and outer pillar cells, along with Deiters’ cells, Hensen’s cells and the inner sulcus cells. In 5 out of 19 cochleae were other areas of the cochlea, such as the endosteal cells lining the perilymphatic spaces and the stria vascularis, observed to express GFP.

#### (c) Long-term NT expression

An antibody to BDNF co-localised with 24 out of 35 GFP-positive cells in sections from Ad-GFP-NTs injected GPs (n = 9) (figure 3) and was not detected when sections were treated with a blocking peptide for BDNF. NT3 expression could not be detected immunohistochemically using commercially available antibodies. In one separate experiment we injected a cochlea with Ad-GFP-NT3 alone and confirmed that this viral vector was able to transduce cells and express GFP, but at a lower intensity than that seen in GPs injected with Ad-GFP-NTs (data not shown).

### 2. Effects of Gene Therapy on SGN Survival and Fibre Regrowth

#### (a) SGN density

Cochleae treated with Ad-GFP-NTs (figure 4a) and examined at T8-wk showed a significantly greater density of SGNs in the treated cochleae compared to the contralateral non-treated cochleae (repeated measures one way ANOVA p<0.005). Post hoc analysis showed significantly greater SGN densities in the basal (1197±76 versus 919±61 SGNs/mm^2^) and middle turns of the cochlea (1239±102 versus 939±104 SGNs/mm^2^) (repeated measures one way ANOVA, post hoc Holm-Sidak, p<0.05). There was no significant difference in SGN density in the apical region of the cochlea between treated and contralateral untreated cochleae. Injection of the control vector, Ad-GFP did not result in a significant difference in SGN density in any region when compared to the contralateral untreated cochleae. Moreover, a direct comparison on the effects of Ad-NT treatment and Ad-GFP treatment was conducted using data normalised to the contralateral control. This analysis showed a significant difference between Ad-NT and Ad-GFP cohorts (one way ANVOA, p<0.05).

Cochleae examined at T12-wk (figure 4b) showed a significantly greater density of SGNs in the treated cochleae with Ad-GFP-NTs compared to the contralateral non-treated cochleae (repeated measures one way ANOVA p<0.001). Post hoc analysis showed a significantly greater density of SGNs in the basal turn of treated cochleae (1248±70 SGNs/mm^2^ vs. 819±101 SGNs/mm^2^) (repeated measures one way ANOVA, post hoc Holm-Sidak, p<0.05). The SGN density was not significantly different in the middle or apical turns of treated cochleae in comparison to contralateral untreated cochleae. Once again, there was no significant difference in the density of SGNs in any regions of the Ad-GFP treated cochleae, compared to the contralateral untreated cochleae. As with the T8-wk cohort, a direct comparison on the effects of Ad-NT treatment and Ad-GFP treatment, examined at T12-wk, was conducted using data normalised to the contralateral control. This analysis showed a significant different between Ad-NT and Ad-GFP cohorts (one way ANVOA, p<0.05).

To ensure that these results were not confounded by a change in soma size after NT-gene therapy treatment, 30 randomly selected soma areas were measured from each treated and untreated contralateral cochlea. There was no significant difference between the treated (averages 205.3 µm^2^±7.2 µm^2^, 198.1 µm^2^±19.3 µm^2^) and untreated (201.3 µm^2^±5.4 µm^2^, 192 µm^2^±12.7 µm^2^) cochleae, measured in the lower basal turn in the T8-wk or T12-wk cohorts, respectively (paired t-test, p = 0.744; p = 0.257).

#### (b) Peripheral fibre resprouting

Examination of peripheral fibres in cochlear surface preparations in Ad-GFP or Ad-GFP-NTs groups revealed growth of significantly more peripheral fibres in the vicinity of NT-expressing cells when compared to GFP-only expressing cells (figure 5a–d).

In the T8-wk cohort, the number of NF-200 positive pixels surrounding the transduced cells in GPs treated with Ad-GFP-NTs (375.2±102.4, n = 22 cells) was significantly greater than the number surrounding the transduced cells from Ad-GFP treated GPs (40.4±14.8, n = 7 cells); p<0.05, t-test.

In the T12-wk cohort, the number of NF-200 positive pixels surrounding the transduced cells in GPs treated with Ad-GFP-NTs (221.5±72.5, n = 9 cells) was significantly greater than the number surrounding the transduced cells in Ad-GFP treated GPs (41.5±15.9, n = 19 cells); p<0.05, Mann-Whitney test.

### 3. Safety of Viral Gene Therapy

#### (a) Risk to residual hearing

To analyse the long-term impact of viral vector injection on residual hearing, hearing thresholds were measured prior to and 11 weeks following injection of the viral vector into the SM of normal hearing GPs. There was a significant shift in frequency-specific auditory brainstem response (ABR) thresholds over the treatment period (pre- to post-injection periods) in the 16, 24 and 32 kHz of the cochlea (figure 6) (paired t-test, p<0.05).

#### (b) Tissue response to viral injections into the scala media

**Figure 7:**
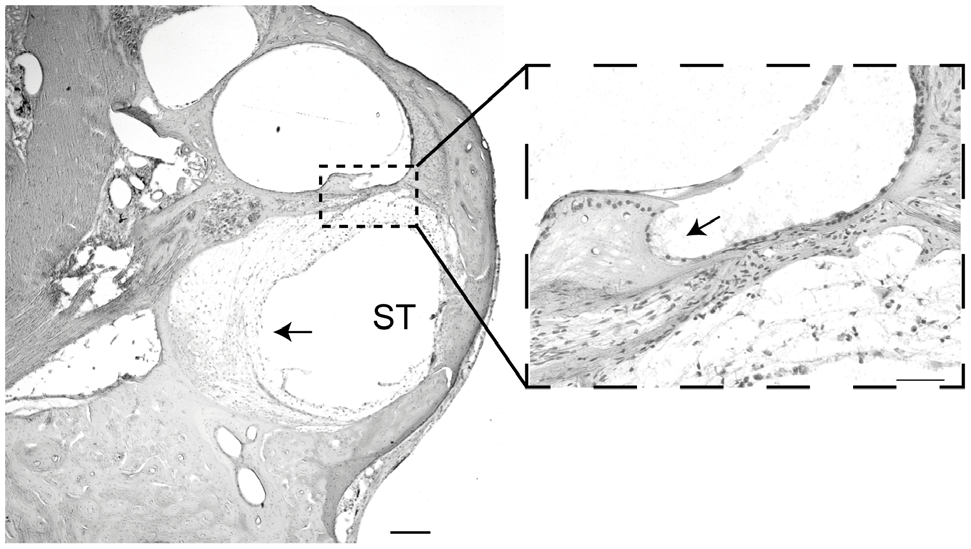
Histological sections showing an example of tissue response in the lower basal turn. Examples of cochlear cross sections illustrate the location and extent of tissue response (arrows) characterised by fibrosis and minimal new bone growth in the lower basal turn of the cochlea associated with viral injection surgery. The tissue response was localised to the basal turn scala tympani (ST) with only a very minor response observed in the scala media (zoomed image). Scale bar = 200 µm, and 50 µm on zoomed imaged.

There was no evidence of multinucleated giant cell or macrophage infiltration into treated cochleae, which are some of the hallmarks of an immunogenic response to a viral pathogen in the cochlea [35]. There was, however, a mild-moderate tissue response, which was most prominent in the lower basal region where the surgery was performed, characterised by fibrosis and new bone growth observed in 10 of 24 treated cochleae (figure 7). The tissue response was quantified by measuring the area of the scala tympani which was occupied by fibrosis and new bone growth (47±9.7%, n = 10 GPs). There were only two cases where a mild response was observed in the upper basal turn. Furthermore, of the 10 injected cochleae that exhibited a tissue response, only one of these was treated with Ad-GFP-NTs. Of the 10 cochleae that exhibited a tissue response in the scala tympani, 5 also displayed a very mild tissue response in the scala media, which was characterised by very thin fibrosis tissue growth, located between the spiral limbus and the OC.

## Discussion

The key finding of this study is that NT gene therapy provides effective and sustained neural protection in the long term deafened cochlea. This study is the first to demonstrate the effectiveness of NT gene therapy for durations of treatment beyond 30 days, even in the much degenerated OC of the deafened GP. NT gene therapy resulted in a significant increase in SGN survival in the basal turn when examined after T8-wk and T12-wk compared to non-injected cochleae, and promoted local peripheral fibre growth towards transduced cells expressing NTs.

### Viral Vector Expression Persists Long-term in the Deafened Cochlea

Deafening using a combined treatment of furosemide and kanamycin in GPs resulted in a rapid degeneration of the OC, with significant degeneration occurring within 1 week in the basal region of the cochlea. This degeneration is progressive and was marked by a loss of HCs and supporting cells, and by a flattening of the sensory epithelia, similar to that found in previous studies [36], [37]. The introduction of BDNF and NT3 via gene therapy did not halt or slow aminoglycoside-induced degeneration of the OC in the basal turn, suggesting that the effects observed in NT-gene therapy treated cochleae are due to a direct effects of NT released by the transfected cells on the SGNs themselves rather than an indirect effect via a better preserved OC. Importantly, however, even in the degenerating OC, viral transduction was possible, indicated by the presence of GFP expression in this region. Furthermore, gene expression persisted for up to 11 weeks post injection, despite continued degeneration of the OC after gene therapy intervention.

The expression was mainly restricted to the basal and middle turn of the GP cochlea, with only rare expression in the apex, a finding that has also been demonstrated in shorter term studies [26], [28]. Similar to the observations in shorter term studies, the cells transduced included the supporting cells of the OC (pillar cells, Deiters’, Hensen’s cells and inner sulcus cells), along with the interdental cells of the spiral limbus [26], [28]. This expression pattern corresponded to the expression pattern of the surface receptors coxsackie Ad receptors (CARs) and α_v_β3/α_v_β5 integrin co-receptors [38], the receptors necessary for Ad transduction [39], [40]. This restricted long-term expression is beneficial for NT gene therapy, as it may allow for survival and directed regrowth of peripheral fibres towards target areas, such as regenerated HCs or the cochlear implant electrode array. It has, however, been shown that in the long-term deafened cochlea (8 weeks) the majority of support cells of the OC are lost, leaving only the interdental cells and cells within the stria vascularis to be transduced [28]. We hypothesise that no survival effect was observed in GPs treated 8 weeks after deafness because of the distance of the transduced cells from the SGNs and a low level of transduction, resulting in a long diffusion distance and a relatively low concentration of NTs at the SGNs. Taken together, these studies suggests that while long-term expression is possible, it is integral to the efficacy of this therapy that the intervention occurs while the supporting cells of the OC are present and are able to be transduced.

In order to achieve a rescue effect, the sustained expression of our reporter, GFP, must be matched with sustained expression of NTs. BDNF expression in Ad-GFP-NTs injected GPs was confirmed by immunohistochemistry, while NT3 expression was not detected despite the confirmed production of NT3 in cell lines as assessed by an ELISA. However, in one additional experiment we injected a GP cochlea with Ad-GFP-NT3 alone and showed GFP expression, suggesting that while the vector encoding for NT-3 was able to enter the cells the antigen produced may be at levels lower than that required for detection by current commercial antibodies. These findings indicate that sustained NT expression is possible in the deafened cochlea when introduced using viral mediated gene transfer.

### Rescue Effects of NT-gene Therapy

Neurotrophin gene therapy elicited a rescue effect, demonstrated by an increase in SGN survival in deafened GPs injected with Ad-GFP-NTs. This increase in SGN survival was observed in the basal turn, when examined at T8-wk and T12-wk. The survival effect also correlated with the pattern of gene expression, with the highest level of survival observed in the basal regions of the cochlea. Also in line with expression data, an increase in SGN survival was observed in the middle turn at T8-wk. Interestingly, a comparison of the T8-wk and T12-wk SGN densities revealed no significant difference between these two time points suggesting that in areas of high gene expression there is a degree of stability in SGN survival over time. Moreover, the SGN density in T8-wk or T12-wk NT-treated deaf GPs was not significantly different to that in normal hearing GPs in the basal turn (data not shown). The middle turn SGN densities of T8-wk NT-treated deaf GPs were also comparable to that in normal hearing GPs (data not shown). Collectively, these data demonstrate, for the first time, that NT gene therapy leads to a localised, sustained expression of NT in the deafened GP cochlea. Importantly this sustained expression is able to protect SGNs in the vicinity of this expression from ongoing degeneration after deafness.

In the deafened GP cochlea, peripheral fibres have been shown to retract [41]. Although there was evidence of subsequent resprouting, the fibres were disorganised with some projecting towards the basilar membrane while others loop back around within the osseous spiral lamina [18], [26]. Disorganised resprouting may reduce the fidelity of a CI that functions by providing electrical stimulation to spatially distinct sub-populations of SGNs [42]. At present the electrode spacing of the CI is much larger than the lateral deviation of resprouting fibres observed following 4 weeks of pump-based NT delivery, and this disorganised sprouting is therefore unlikely to have functional consequences [42]. However, with the advent of new electrode design and stimulation strategies, these small deviations may limit any improvement in the precision of neural activation. The localised production of NTs by supporting cells of the OC in GPs treated with NT-gene therapy, however, maintained and directed the resprouting peripheral fibres in the T8-wk and T12-wk cohorts. This was indicated by the presence of significantly more fibres near transfected cells expressing NTs in the basal turn. Reducing the distance between the peripheral fibre and stimulating electrode would be expected to reduce electrical thresholds [43], moreover enhanced SGN survival may also encourage improved electrode design and stimulation strategies that can deliver more focused neural excitation [44], providing higher resolution of spatial and temporal information to the auditory pathway.

### Safety and Clinical Implications

The need to protect residual hearing during cochlear implantation, and by extension during any adjunct therapy, has become increasingly important as a greater number of people with residual hearing are undergoing cochlear implantation. To assess the effects of viral mediated gene therapy delivery to the basal turn SM, hearing thresholds were examined in normal hearing GPs prior to and 11 weeks post Ad-GFP injection. There was a significant decrease in hearing sensitivity in the 16–32 kHz region of the cochlea. This region of the cochlea corresponds to the basal turn and the site of injection. There was also a threshold shift of 20 dB in the 1 kHz region (apex) of the cochlea, however, this loss was not statistically significant. The increase in hearing threshold observed in this study may result from the vibrations or noise of the surgical drill or from the opening of the cochlea, which has been previously reported [31]. It may also occur as a result of the piercing of the basilar membrane and injection into the SM, or a combination of all these factors. Whilst this decrease in hearing sensitivity appears to persist long-term, the affected regions are generally localised to the higher frequencies, which in a clinical setting are usually already significantly damaged prior to intervention.

As with the introduction of any viral based vector into the body, it is necessary to determine the extent of an immune response. In the current study, the injection of the viral vector into the SM was associated with a tissue response characterised by loose fibrotic tissue and, in some instances, new bone growth, with the response typically being localised to the scala tympani of the basal turn. A similar tissue response is commonly observed after a cochleostomy, due to fine bone fragments entering the scala tympani during the drilling [45]. A very mild fibrotic response was also observed in the SM of the basal turn. Importantly there was no indication of infiltration of multi-nucleated giant cells or macrophages into the cochlea, which would be indicative of a chronic immune response [35]. Taken together, these observations suggest that the viral vector does not cause a direct immune response but rather that the surgery associated with accessing the cochlea causes an inflammatory tissue response. The lack of immune response may be due to the blood-labyrinth barrier, which confers a level of immunoprotection to the cochlea, as well as to improvements in the safety of new generation Ad vectors [46], [47].

The results of the present study support several findings, which are integral to the clinical viability of Ad-mediated NT gene therapy: ability to have sustained expression resulting in protection of SGNs, including protection and regrowth of peripheral fibres, and no reactive immune response. Therefore neurotrophin gene-therapy could be considered as a potential adjunct to a cochlear implantation. As such the viral injection would ideally be performed during CI surgery. The surgical access to the endolymphatic space can be achieved in a number of ways in humans: by passing a micropipette apically through the round window, or by performing a cochleostomy anteriorly to the round window. However these are challenging procedures. The round window approach, for example, is constrained by the posterior tympanotomy, more specifically the access from the mastoid to the middle ear is between the facial nerve and the chorda tympani. In future studies, it will important to determine the efficacy of combining cochlear implantation with NT gene therapy both from a surgical and a functional perspective.

This study has demonstrated that NT gene therapy offers a strategy for long-term SGN preservation and directed fibre regrowth with a single intervention. These results indicate that gene therapy may be a useful technique in the treatment of other neurological disorders as it allows for the localised, targeted, stable and efficient introduction of a gene or genes. Neuroprotective and regenerative therapies using trophic factors, such as NTs, have garnered much attention of late, particularly in the areas of Parkinson’s disease, retinitis pigmentosa and spinal cord injury, just to name a few [48], [49], [50]. Initial clinical studies involving Parkinson’s disease patients, for example, used a mechanical pump to infuse glial cell-derived neurotrophic factor (GNDF) into the lateral ventricles. However, these trails failed to deliver positive patient outcome [51]. The lack of a therapeutic outcome may be due to the inability of the GDNF peptide to reach the target tissues. The failures of these clinical trials have led to the exploration of alternative methods for delivering GDNF to target brain regions, such as the use of viral vectors. The delivery of GDNF using viral vectors has initially been trialled in non-human primates; these trials have yielded positive results, with an increase in dopaminergic function up to 6 months post inoculation and clinical improvements without adverse effects [50]. These findings, along with those presented in this study, demonstrate the effectiveness of NT gene therapy in treating a number of neurodegenerative diseases, and while further pre-clinical and clinical studies are needed, such treatments hold real promise to better patient outcomes.
